# Toward Graph-Based Decoding of Tumor Evolution: Spatial Inference of Copy Number Variations

**DOI:** 10.3390/diagnostics15243169

**Published:** 2025-12-12

**Authors:** Yujia Zhang, Yitao Yang, Yan Kong, Bingxu Zhong, Kenta Nakai, Hui Lu

**Affiliations:** 1 SJTU-Yale Joint Center for Biostatistics and Data Science, State Key Laboratory of Microbial Metabolism, Joint International Research Laboratory of Metabolic and Developmental Sciences, Department of Bioinformatics and Biostatistics, School of Life Sciences and Biotechnology, Shanghai Jiao Tong University, Shanghai 200240, China; yukizhang@sjtu.edu.cn (Y.Z.); zhong_bx@sjtu.edu.cn (B.Z.); 2 Department of Computational Biology and Medical Sciences, Graduate School of Frontier Sciences, University of Tokyo, Tokyo 113-0033, Japan; yang-yitao@g.ecc.u-tokyo.ac.jp; 3 National Center for Translational Medicine, Shanghai Jiao Tong University, Shanghai 200240, China; kongyan@sjtu.edu.cn; 4 Shanghai-Chongqing Artificial Intelligence Research Institute, Chongqing 401300, China; 5 Human Genome Center, Institute of Medical Science, University of Tokyo, Tokyo 108-0071, Japan; 6 Shanghai Engineering Research Center for Big Data in Pediatric Precision Medicine, Shanghai Children’s Hospital, School of Medicine, Shanghai Jiao Tong University, Shanghai 200240, China; 7 Institute of Bioinformatics, Shanghai Academy of Experimental Medicine, Shanghai 200240, China

**Keywords:** spatial transcriptomics, tumor evolution, tumor heterogeneity, multi-omics, graph neural networks, copy number variation

## Abstract

**Background/Objectives:** Constructing a comprehensive spatiotemporal map of tumor heterogeneity is essential for understanding tumor evolution, with copy number variation (CNV) being a significant feature. Existing studies often rely on tools originally developed for single-cell data, which fail to utilize spatial information, often leading to an incomplete map of clonal architecture. Our study aims to develop a model that fully leverages spatial omics data to elucidate spatio-temporal changes in tumor evolution. **Methods:** Here, we introduce SCOIGET (Spatial COpy number Inference by Graph on Evolution of Tumor), a novel framework using graph neural networks with graph attention layers to learn spatial neighborhood features of gene expression and infer copy number variations. This approach integrates spatial multi-omics features to create a comprehensive spatial map of tumor heterogeneity. **Results:** Notably, SCOIGET achieves a substantial reduction in error metrics (e.g., mean squared error, cosine similarity, and distance measures) and produces superior clustering performance, as indicated by higher Silhouette Scores compared to existing methods, validated by both simulated data with spot-level ground truth and patient cohorts. Our model significantly enhances the accuracy of tumor evolution depiction, capturing detailed spatial and temporal changes within the tumor microenvironment. It is versatile and applicable to various downstream tasks, demonstrating strong generalizability across different spatial omics platforms, including 10× Visium and Visium HD and various cancer types, including colorectal cancer and prostate cancer. This robust performance improves research efficiency and provides valuable insights into tumor progression. **Conclusions**: SCOIGET offers an innovative solution by integrating multiple features and advanced algorithms, providing a detailed and accurate representation of tumor heterogeneity and evolution, aiding in the development of personalized cancer treatment strategies.

## 1. Introduction

Tumor evolution, also known as carcinogenesis, is a complex, multi-step process characterized by dynamic changes in morphology, genetic composition, and epigenetic profiles [1]. Studies have demonstrated significant differences in genetic homogeneity and heterogeneity within tumors, with pronounced variations observed between malignant regions and adjacent benign tissues [2,3]. Copy number variations (CNVs), which refer to the gains or losses of specific genomic regions, are crucial for evaluating such heterogeneity and play vital roles in influencing cell proliferation, therapeutic response, and resistance mechanisms [4,5]. Traditionally, whole-genome and whole-exome sequencing have been the primary approaches for identifying CNVs, providing a broad overview of genomic alterations based on next-generation sequencing (NGS) data [6,7,8]. However, these “bulk” approaches fail to capture intratumoral heterogeneity, particularly spatial heterogeneity, which is crucial for understanding tumor progression and developing targeted therapies. Although spatially resolved DNA sequencing could theoretically address this gap [9], its practical application is limited by the scarcity and high cost of such data.

Recent studies have revealed a strong correlation between CNVs and differential gene expression at the RNA level [10], establishing a clear biological premise for inferring genomic changes from transcriptomic data. This principle, that “large CNVs leave a signature in gene expression”, is the foundation for numerous successful CNV inference tools developed for single-cell RNA sequencing (scRNA-seq) data [11,12,13,14]. For instance, InferCNV [11] identifies chromosome-level CNVs by comparing the gene expression profiles of target cells to a reference population of normal cells, but its reliance on well-defined normal references limits its applicability. CopyKAT [12] and SCEVAN [13] address this issue by using unsupervised clustering to distinguish tumor cells from normal cells, but their probabilistic frameworks are constrained when handling large or highly heterogeneous datasets. CopyVAE [14], which applies a variational autoencoder (VAE) to capture nonlinear gene expression patterns for CNV inference, depends heavily on the accurate identification of diploid cells, which can be challenging in diverse tumor contexts. Furthermore, all these scRNA-seq-based methods overlook spatial information, making them inadequate for addressing spatial heterogeneity within tumors. This limitation prevents these approaches from effectively modeling the complex spatial dynamics that are critical for understanding tumor evolution.

Recognizing the insufficiency of spatial information in existing methods, studies in spatial transcriptomics (ST) have underscored the indispensable role of spatial context in accurately mapping gene expression patterns [15,16]. The recently proposed CalicoST [17] method utilizes hidden Markov models (HMM) to detect correlations within genomic intervals and hidden Markov random fields (HMRF) to model spatial correlations between cancer clones. While this approach integrates spatial data, it assumes clone state consistency across neighboring spatial regions, a strong constraint that can lead to inaccurate predictions in tumors with high spatial heterogeneity. Additionally, for low-coverage ST data, CalicoST aggregates neighboring spot counts to improve robustness, but this sacrifices spatial resolution and limits its generalizability across different platforms. Moreover, the reliance on computationally intensive statistical optimization algorithms, such as negative binomial and beta-binomial distributions, results in lengthy runtimes (2–8 h), further constraining its scalability and applicability in large-scale studies.

To address these limitations, we propose SCOIGET (Spatial COpy number Inference by Graph on Evolution of Tumor), a novel framework that leverages spatial omics data to achieve accurate and efficient CNV inference. The key innovation of SCOIGET lies in its use of graph structures to dynamically model spatial relationships, enabling the method to capture local spatial heterogeneity and adapt to tumors with varying complexity. By incorporating graph neural networks (GNN) with graph attention (GAT) mechanisms, SCOIGET can flexibly adjust the influence of neighboring spots, overcoming the rigidity of uniform neighbor treatment. Additionally, its graph-based design allows information from low-coverage regions to propagate through adjacency relationships, minimizing the need for spot aggregation while preserving fine spatial resolution. These features ensure the robust performance of our model across diverse tumor samples and sequencing platforms. Unlike computationally intensive statistical optimizations, SCOIGET employs deep learning strategies, offering high computational efficiency and scalability for large datasets. This comprehensive integration of spatial and transcriptomic data, coupled with its adaptability and efficiency, makes SCOIGET a powerful tool for exploring tumor evolution, uncovering tumor clone dynamics, and supporting early therapeutic interventions.

## 2. Materials and Methods

### 2.1. Dataset Information

We utilized four spatial transcriptomics datasets, including three colorectal cancer (CRC) cohorts and one prostate cancer (PCa) cohort. The first CRC Visium dataset was obtained from the HTAN-WUSTL atlas [18], accompanied by whole-exome sequencing (WES) data used to construct the gold standard for copy number analysis. The second dataset was derived from a Cell publication in 2023 [19], consisting of four CRC Visium samples from the same patient covering different stages.

The third dataset comprises three CRC Visium HD samples. Specifically, we utilized the curated ‘Visium HD Human Colorectal Cancer (FFPE)’ dataset hosted on Zenodo (https://zenodo.org/records/11402686) [20]. Finally, the fourth dataset was a prostate cancer Visium dataset from a 2022 Nature study [3], comprising four samples collected from a single patient.

The first dataset served as a validation set, and the remaining three were used as case studies to explore the utility and generalizability of our method. Further details are provided in Appendix A.1 and Table A1.

### 2.2. Construction of Pseudo-Single-Cell Resolution for Visium HD

To ensure biological relevance at the cellular level, we utilized a pseudo-single-cell resolution approach rather than analyzing raw Visium HD bins. As detailed in the dataset documentation provided by Kiessling et al. [20] (SpaceHack 2023), this resolution was achieved through a rigorous segmentation and aggregation workflow:

1. Nuclei Segmentation: The accompanying high-resolution H&E histological images were segmented to delineate nuclear boundaries using Napari.
2. Spatial Aggregation: The SpatialData framework [21] was utilized to establish a common coordinate system, aligning the 8 µm Visium HD bins with the segmented nuclear geometries. Bins spatially overlapping with these nuclear boundaries were then computationally aggregated to generate image-segmented pseudo-single-cell expression profiles.
3. Validation: The biological validity of these pseudo-cells was confirmed through expert pathologist annotations provided within the dataset. These annotations, based on H&E staining and characteristic marker gene expression, served as the ground truth to benchmark the spatial domains identified by SCOIGET.

### 2.3. Data Preprocessing

All datasets, generated using the 10× Genomics Visium [22] and Visium HD [23] platforms, were processed through a standardized pipeline. Spatial transcriptomics data, including gene expression matrices, spatial coordinates, and high-resolution tissue images, were integrated into AnnData objects using Python Scanpy library [24] and the BMAP platform [25]. Quality control involved filtering out cells with fewer than ten total counts and genes expressed in fewer than five spots. Cell cycle-related genes, mitochondrial genes, and human leukocyte antigen (HLA) genes were removed to minimize non-tumor-related variability. Gene expression counts were normalized to ensure a consistent total count per cell, log-transformed to stabilize variance, and scaled to reduce the influence of outliers. Diagnostic visualizations, such as histograms of QC metrics, UMAP embeddings, and spatial scatter plots, were generated to assess data integrity and spatial distributions.

### 2.4. Sample Integration

To integrate samples from different stages into a unified analysis, we employed the Harmony algorithm [26] to eliminate batch effects. Using the Python 3.8.20 implementation of Harmony, harmonypy, we integrated ST data stored in an AnnData object. This process was conducted after PCA computation and before constructing the neighbor graph, ensuring effective removal of batch effects while preserving biological variation. The resulting batch-corrected embedding was used for downstream analyses, providing a harmonized dataset with minimal technical biases.

### 2.5. Gene Annotation and Binning

To map gene expression data to specific genomic regions, we annotated each gene using the Ensembl genome database (Release 98). Each gene was linked to its chromosome and genomic coordinates (start and end positions) based on its Ensembl gene ID. Genes lacking valid chromosomal information were excluded to ensure data accuracy.

To reduce data sparsity and improve CNV detection, a gene binning strategy was employed. Genes were first sorted by chromosome and genomic position, then grouped into bins of 25 adjacent genes. For chromosomes where the total number of genes was not divisible by 25, lowly expressed genes were excluded to align the count. Within each bin, gene expression values were aggregated by summing, producing a condensed dataset that preserves critical genomic information while reducing noise and dimensionality.

To reduce data sparsity while retaining genomic position, a gene binning strategy was employed. Genes were first sorted by chromosome and genomic position, and this process was applied independently to each chromosome. Genes were grouped into non-overlapping bins of 25 adjacent genes. This bin size was chosen empirically as it provides a balance between resolving localized CNV events and smoothing expression noise. For chromosomes where the total number of genes was not divisible by 25, the final incomplete bin was excluded. While this binning strategy does not explicitly model complex genomic rearrangements (e.g., translocations), the HMM component (see Section 2.6) remains capable of detecting the resulting abrupt CNV state changes between adjacent genomic bins.

### 2.6. Spatial Graph Construction

To integrate spatial relationships with gene expression data, we constructed spatial neighbor graphs for each phase, iteratively refining both spatial and genomic features.

#### 2.6.1. Initial Graph Construction

In the first phase, a k-nearest neighbors (k-NN) [27] graph was built using binned gene expression data and spatial coordinates. Each spot was connected to its five nearest neighbors based on spatial Euclidean distance, forming the graph’s adjacency matrix. Edge weights were computed by combining spatial proximity and expression similarity. Binned expression data were standardized and reduced via principal component analysis (PCA) to 32 dimensions to extract informative features. In this step, the k-NN connections were established based on PCA-transformed Euclidean distances in the feature space. Softmax normalization was then applied to convert these distances into edge probabilities, emphasizing stronger connections between nodes with similar expression profiles while incorporating spatial neighborhood information.

#### 2.6.2. Refined Graph Construction

In the second phase, pseudo-CNV estimates from the initial round of training were incorporated as new node features, augmenting the graph with genomic information. These updated features, which combined spatial and refined genomic characteristics, were further standardized and reduced via PCA to 32 dimensions. A new k-NN graph was constructed using the PCA-transformed feature space, ensuring that edge connections reflected both spatial proximity and genomic feature similarity. This iterative refinement progressively enhanced the spatial representation, improving CNV localization accuracy and spatial clone identification precision. The edge weights in this refined graph were recalculated using the updated feature embeddings, and softmax normalization was reapplied to ensure probabilistic interpretation while maintaining biologically relevant connectivity patterns.

### 2.7. SCOIGET Framework

The core of the SCOIGET framework is a graph neural network (GNN) [28] with graph attention layers, which specifically designed to capture complex spatial-transcriptomic-genomic interactions within spatial transcriptomics data. The model integrates gene expression profiles with spatial information to detect CNVs and infer tumor heterogeneity.

1. Input Data: SCOIGET utilizes three main components: (1) Node Features, where each spot or cell is represented by either binned gene expression data (feat) or pseudo-copy number profiles (norm_x) depending on the training stage; (2) Spatial Graph Structure, which encodes spatial relationships in an adjacency matrix (graph_neigh) where nodes represent spots or cells and edges indicate spatial proximity based on tissue architecture; and (3) Edge Attributes, which quantify the similarity between neighboring spots by calculating gene expression distances and normalizing them using softmax.
2. Model Architecture: SCOIGET’s model comprises three primary components: an Encoder, a Decoder, and a Copy Number Encoder (CNEncoder). The Encoder utilizes three Graph Attention Network (GAT) layers [29] with multiple attention heads and ReLU activations to learn latent representations that capture complex spatial–transcriptomic–genomic interactions. The Decoder reconstructs the original input features from these latent representations through fully connected layers, including an intermediate layer with 128 units and ReLU activations to preserve intricate patterns. The CNEncoder estimates CNVs from the reconstructed features by employing a Hidden Markov Model (HMM) [30], which models genomic bins with discrete states and Gaussian emission probabilities. This encoder integrates spatial smoothing to enhance CNV localization and includes a regularization loss term to prevent overfitting.
3. Copy Number Estimation and Refinement: The CNEncoder in SCOIGET estimates CNVs by identifying regions with consistent copy number states through a Hidden Markov Model (HMM). This process involves predicting hidden states that correspond to different copy number levels and applying spatial smoothing to reduce noise and improve CNV localization. The final CNV estimates are normalized and rescaled to ensure consistency across samples. Detailed implementation and parameter settings of the HMM are presented in Appendix A.3.
4. Training Procedure: The model undergoes two phases of training. In the first phase, it uses binned gene expression data and a spatial graph constructed from the original features. The model is trained without a validation set to learn initial latent representations and reconstruct the input features. Following this phase, the CNEncoder estimates pseudo-copy numbers using an HMM, generating initial CNV predictions. In the second phase, the pseudo-copy numbers are incorporated into the node features, and a new spatial graph is created. The model is retrained on this updated graph, utilizing a validation set to monitor performance and prevent overfitting, with early stopping based on validation loss. The CNEncoder re-estimates the copy numbers, refining the CNV profiles with the updated model.

Details of the model can be found in Appendix A.2.

### 2.8. Loss Function

The overall loss function includes several components to ensure effective training.

1. Reconstruction Loss: This measures the discrepancy between the original and reconstructed features, encouraging the model to retain essential information in the latent space:
  (1) $$L_{recon}=\frac{1}{N}\sum_{i=1}^{N}{\left|x_{i}-\hat{x_{i}}\right|}^{2}$$
2. KL Divergence Loss: This regularizes the latent space by encouraging the learned distribution to be close to a prior distribution, which prevents overfitting and ensures meaningful latent representations:
  (2) $$L_{KL}=\frac{1}{2}\sum_{i=1}^{N}\left(\mathrm{Tr}\left(\Sigma_{i}\right)+\mu_{i}^{⊤}\mu_{i}-\log\det\left(\Sigma_{i}\right)-d\right)$$
  where $\mu_{i}$ and $\Sigma_{i}$ are the mean and covariance of the latent distribution for sample i, and d is the dimensionality of the latent space.
3. Regularization Loss: This uses L2 regularization on the reconstructed features helps prevent overfitting and encourages smooth predictions:
  (3) $$L_{reg}=\lambda \sum_{i=1}^{N}{\left|\hat{x_{i}}\right|}^{2}$$
  where λ controls the contribution of the regularization term.
4. Spatial Smoothing Loss: This enforces spatial consistency in the predicted copy numbers by minimizing the discrepancy between the copy numbers of connected nodes in the graph:
  (4) $$L_{smooth}=\frac{1}{\left|E\right|}\sum_{\left(i,j\right)\in E}{\left|{\mathrm{norm}\text{\_}\mathrm{copy}}_{i}-{\mathrm{norm}\text{\_}\mathrm{copy}}_{j}\right|}^{2}$$
  where E is the set of edges in the graph, and $\left|E\right|$ is the number of edges.
5. The Total Loss is a weighted sum of the reconstruction loss, *KL* divergence loss, regularization loss, and spatial smoothing terms:
  (5) $$L_{total}=L_{recon}+\beta \cdot L_{KL}+\lambda {\cdot L}_{reg}+\gamma \cdot L_{smooth}$$

### 2.9. Model Implementation and Training

The model is implemented using PyTorch 2.2.2 and PyTorch Geometric (PyG) 2.5.2 [31] libraries, leveraging GPU acceleration for improved performance. Training is conducted with the Adam optimizer, typically set with a learning rate of 0.001. During each epoch, the model performs forward propagation to compute outputs and loss, followed by backpropagation to update the model parameters. In the first training phase, the focus is on learning meaningful latent representations without the use of a validation set. In the second phase, a validation set (20% of the data) is introduced to monitor model performance and guide training adjustments, with early stopping employed if the validation loss does not improve.

All models were trained using an NVIDIA A100 GPU with 80 GB of memory. For a typical validation dataset (e.g., HT260C1, ~4000 spots), the entire two-phase training procedure required approximately 35 min of computation time, demonstrating the model’s computational efficiency.

### 2.10. Baseline Methods

To evaluate the performance of SCOIGET, we compare it with four existing CNV inference algorithms: InferCNV [11], CopyVAE [14], CopyKAT [12], and SCEVAN [13]. InferCNV estimates CNVs by comparing gene expression levels between tumor and reference normal cells, employing a sliding window approach to smooth the expression signals. CopyVAE, a variational autoencoder-based method, infers CNVs from single-cell RNA-seq data, capturing the probabilistic nature of CNV states. CopyKAT segments tumor cells based on gene expression levels to identify large-scale CNVs, effectively distinguishing tumor cells from normal ones. SCEVAN integrates spatial and genomic information to infer CNVs, leveraging advanced statistical models to improve detection accuracy. To ensure reproducibility and fairness, all baseline algorithms were executed using specific parameters derived from their official recommendations for spatial/single-cell data. Detailed parameter settings and software versions are listed in Table A2. Specifically: InferCNV [11] was run with cutoff = 0.1 and HMM enabled; CopyKAT [12] used KS.cut = 0.1; SCEVAN [13] utilized the standard unsupervised pipeline with beta_vega = 0.5; CopyVAE [14] was trained with a latent dimension of 32 for 50 epochs. All methods used the same preprocessed gene expression matrix as input. Since CalicoST requires raw sequencing files for computation, we did not include it in the comparison here due to data availability constraints. WES data was used as an independent benchmark for evaluating the accuracy of large-scale CNV inference. As bulk WES reflects an average signal from the entire tissue, it cannot serve as a ‘gold standard’ for spot-level predictions. However, it provides robust orthogonal validation for assessing whether the inferred CNV profiles correctly capture broad, chromosome-arm-level gains and losses.

### 2.11. Evaluation Metrics

The performance of SCOIGET and the contrast algorithms is comprehensively evaluated using several metrics. The Mean Squared Error (MSE) measures the average squared difference between predicted and true copy number values, with WES data serving as the baseline. Cosine Similarity evaluates the similarity between CNV profiles by comparing them as high-dimensional vectors, with values closer to 1 indicating higher similarity. Euclidean Distance quantifies the straight-line distance between predicted and true CNV values, while Manhattan Distance calculates the absolute distance between these values. The Silhouette Score assesses clustering performance by measuring intra-cluster cohesion and inter-cluster separation, with higher values (closer to 1) indicating well-defined and well-separated clusters (Appendix A.4).

### 2.12. Simulation Study Design

To generate a ground truth dataset for spot-level validation, we simulated a 40 × 40 spatial grid (1600 spots) with three predefined spatial clones (one Normal, one Gain-heavy, one Loss-heavy). We utilized gene locations from the standard 10× Visium mouse reference. The gene expression count matrix was generated by applying discrete CNV changes (e.g., +1 for gain regions, −1 for loss regions) to the log-normalized expression, followed by the introduction of Poisson noise to simulate the sparsity and noise characteristic of real spatial transcriptomics data. The specific CNV regions and noise parameters were fixed to ensure reproducibility and to establish a precise spot-level ground truth for direct quantitative comparison.

### 2.13. Spatial Domain Identification

Copy number features were clustered using the Leiden algorithm [32], which optimizing modularity to identify communities in graphs. The modularity function is defined as

(6) $$\mathrm{Modularity}=\frac{1}{2m}\sum_{i,j}\left(A_{ij}-\frac{k_{i}k_{j}}{2m}\right)\delta \left(c_{i},c_{j}\right)$$

where $A_{ij}$ is the adjacency matrix, $k_{i}$ and $k_{j}$ are the degrees of nodes i and j, m is the total number of edges, and $\delta \left(c_{i},c_{j}\right)$ equals 1 if nodes i and j are in the same community, and 0 otherwise. This approach segments the tissue into distinct spatial domains, corresponding to different tumor clones.

### 2.14. Tumor Evolution Pattern Inference

The ultimate goal of SCOIGET is to infer tumor evolution patterns. High-dimensional embeddings capture both spatial and genomic features of the tumor microenvironment, providing a comprehensive representation of tumor dynamics. Tumor clones are identified based on clustering results, and their evolutionary relationships are analyzed through Partition-based Graph Abstraction (PAGA) [33], which constructs a phylogenetic tree to visualize the progression and diversification of tumor clones. This evolutionary tree enhances our understanding of tumor development and metastasis.

### 2.15. Survival Analysis

To evaluate the prognostic significance of differentially expressed genes identified among subclones by the SCOIGET algorithm, we performed a survival analysis using the GEPIA2 web server [34] (http://gepia2.cancer-pku.cn/) with data from the TCGA-COAD (Colon Adenocarcinoma) cohort. Specifically, the genes deemed differentially expressed between subclones were used to stratify patients into high- and low-expression groups, based on the median expression value (Group Cutoff = Median). Overall Survival (OS) was chosen as the clinical endpoint. GEPIA2 then generated Kaplan–Meier survival curves for each gene, and statistical significance was assessed using the log-rank test.

## 3. Results

### 3.1. Overview of SCOIGET

We introduce SCOIGET, a novel framework designed to infer copy number variations (CNVs) from spatial omics data by leveraging graph neural networks (GNNs) with graph attention layers (GAT). By integrating spatial gene expression profiles with multi-omics features, SCOIGET constructs a comprehensive spatial map of tumor heterogeneity, offering valuable insights into the underlying evolutionary processes.

**Figure 1:**
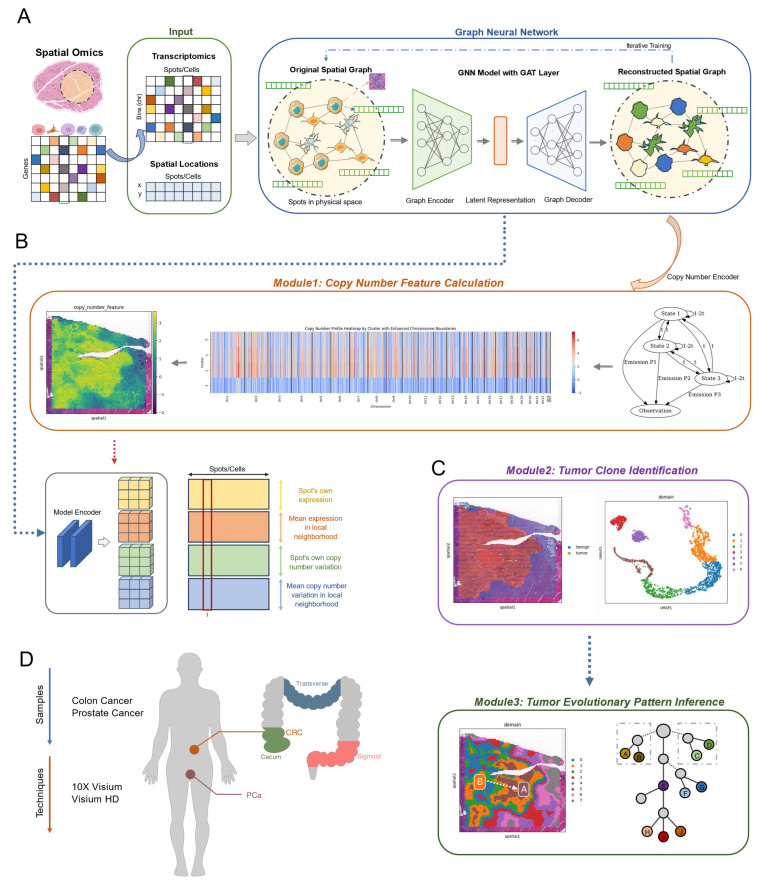
Overview of the SCOIGET framework. (**A**) Model architecture. The SCOIGET framework is built on a graph neural network (GNN) with Graph Attention Network (GAT) layers. It integrates spatial transcriptomics data with genomic features to infer copy number variations (CNVs) and detect tumor heterogeneity. The architecture includes an encoder that learns spatial–transcriptomic–genomic representations, a decoder that reconstructs input features, and a CNEncoder that estimates CNVs using a Hidden Markov Model (HMM). The dashed frame highlights the core GNN module involving iterative feature learning and reconstruction. (**B**) Module 1: Copy number feature calculation. This module computes CNV features by predicting copy number profiles from spatial transcriptomics data, using the CNEncoder and HMM to identify and refine CNVs across the tissue. Spatial smoothing is applied to improve the localization of CNV segments. (**C**) Module 2: Tumor clone identification. Module 3: Tumor evolutionary pattern inference. Module 2 identifies spatial clones by clustering regions with similar genomic and transcriptomic characteristics. Module 3 infers tumor evolutionary patterns, reconstructing clonal dynamics and spatial-temporal tumor progression based on CNV profiles and clustering results. In the evolutionary tree diagram (right), letters A–J represent distinct tumor subclones or evolutionary states identified by the model. (**D**) Dataset information. The datasets used for evaluation include four spatial transcriptomics cohorts: three colorectal cancer (CRC) datasets (HTAN-WUSTL [18], Cell 2023 [19], and 10× Genomics [20]) and one prostate cancer (PCa) dataset (Nature 2022 [3]). These datasets were used for model validation and case studies, with matched WES data providing the gold standard for CNV estimation. Detailed dataset information is provided in Table A1.

The architecture of SCOIGET consists of several key components. The input layer combines transcriptomic profiles with spatial coordinates to build a spatial graph, encoding the neighborhood relationships between cells or tissue regions (Figure 1A). This is followed by the core model, which employs an Encoder–Decoder structure. The encoder uses GAT layers to extract meaningful features from the graph, while the decoder reconstructs these features, preserving both spatial and transcriptional information. In addition, the CNEncoder integrates a Hidden Markov Model (HMM) to detect CNV segments, capturing chromosomal alterations that are critical for understanding tumor evolution (Figure 1B).

SCOIGET supports a variety of downstream analyses through three main modules. The first module calculates CNV features, providing quantitative measures of genomic alterations (Figure 1B). The second module identifies spatial clones by grouping tissue subpopulations with similar genomic and transcriptomic profiles. Finally, the third module infers tumor evolutionary patterns, enabling the reconstruction of clonal dynamics and providing insights into the spatial and temporal progression of the tumor (Figure 1C). Together, these modules position SCOIGET as a powerful and versatile tool for exploring spatial tumor heterogeneity and advancing personalized oncology strategies.

### 3.2. Validation of SCOIGET on Simulated Data

To rigorously address the concern regarding the resolution mismatch of bulk WES validation and to provide direct evidence of SCOIGET’s spot-level accuracy, we conducted a comprehensive simulation study. We generated a synthetic spatial transcriptomics dataset (40 × 40 grid, 1600 spots) with pre-defined ground truth (GT) CNV profiles and three distinct spatial clonal domains (see Section 2.11 for simulation details).

**Figure 2:**
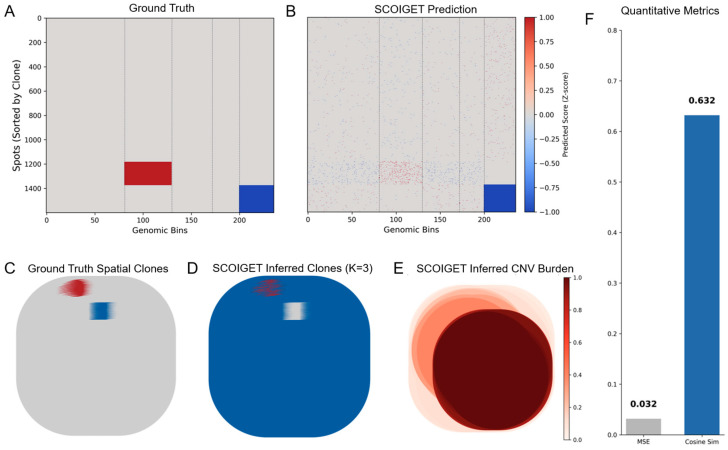
Validation of SCOIGET on Simulated Spatial Transcriptomics Data. (**A**) Heatmap of the ground truth (GT) CNV profiles, sorted by clone identity. (**B**) Heatmap of SCOIGET’s predicted CNV scores, showing the reconstructed CNV patterns. (**C**) Spatial visualization of the ground truth clonal architecture, showing the predefined spatial domains (Normal: Grey, Gain: Red, Loss: Blue). (**D**) Spatial visualization of SCOIGET-inferred clones (K = 3), demonstrating accurate recovery of the GT spatial domains. (**E**) Spatial map of inferred CNV burden, highlighting regions with the highest CNV intensity. (**F**) Quantitative performance metrics, including a high Cosine Similarity of 0.632 and a low Mean Squared Error (MSE) of 0.032, confirming the model’s robust predictive accuracy on spot-level CNV inference.

SCOIGET was applied to this noisy synthetic data to infer the spatial CNV patterns and clonal architecture. The results, summarized in Figure 2, demonstrate high fidelity to the GT. Quantitatively, the predicted CNV profiles achieved an excellent Cosine Similarity of 0.632 and a very low Mean Squared Error (MSE) of 0.032 when compared to the GT CNV matrix (Figure 2A,B,F).

Critically, the spatially inferred clonal domains (Figure 2D) show high concordance with the GT spatial architecture (Figure 2C), confirming that SCOIGET successfully leveraged spatial smoothing to accurately delineate the clonal boundaries. Furthermore, the inferred CNV burden map (Figure 2E) clearly highlights the genetically unstable regions corresponding to the Gain/Loss clones, demonstrating the biological interpretability of our derived features. This simulation provides robust, quantitative validation that SCOIGET accurately recovers spot-level CNV profiles and clonal labels.

### 3.3. SCOIGET Integrates Spatial Omics Features Within a Unified Framework and Accurately Detects Copy Number Features Spatially

To assess the accuracy of CNV inference, we evaluated SCOIGET on eight colorectal cancer (CRC) samples from the HTAN-WUSTL dataset (including HT260C1, HT112C1 [U1, U2], and HT225C1 [U1–U5]). The CNV profiles inferred by SCOIGET were benchmarked against matched whole-exome sequencing (WES) data and compared with four established CNV inference methods (InferCNV, CopyVAE, CopyKAT, and SCEVAN).

**Figure 3:**
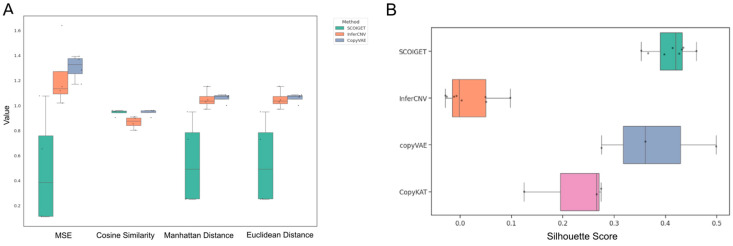
Model accuracy and comparison. (**A**) Performance metrics for CNV inference. The accuracy of SCOIGET’s CNV predictions was assessed using four metrics: Mean Squared Error (MSE), Cosine Similarity, Euclidean Distance, and Manhattan Distance. MSE quantifies the average squared difference between predicted and true CNV values (using WES data as the baseline). Cosine Similarity measures the similarity between predicted and true CNV profiles as high-dimensional vectors, with values closer to 1 indicating higher similarity. Euclidean and Manhattan distances capture the straight-line and absolute differences, respectively, between predicted and true values. SCOIGET outperformed other methods across all metrics, achieving the best overall performance. (**B**) Clustering performance. The Silhouette Score was used to evaluate clustering performance based on the CNV features inferred by SCOIGET. Higher Silhouette Scores indicate better-defined clusters with greater intra-cluster cohesion and inter-cluster separation. SCOIGET-derived CNV features resulted in the highest Silhouette Score, demonstrating superior clustering performance compared to other methods.

As shown in Figure 3A, SCOIGET consistently outperformed alternative methods across several quantitative metrics, including mean squared error (MSE), cosine similarity, Euclidean distance, and Manhattan distance. For example, in sample HT260C1, SCOIGET achieved an MSE of 1.0764, a cosine similarity of 0.903, and both average Manhattan and Euclidean distances of 0.949. In contrast, InferCNV yielded an MSE of 1.6386, a cosine similarity of 0.8525, and distance metrics exceeding 1.15. Similarly, in samples HT112C1-U1 and HT112C1-U2, SCOIGET reported MSE values of 0.1098 and 0.1149, with cosine similarities of 0.9536 and 0.9598, respectively—significantly better than those of the competing methods. Overall, these statistical evaluations indicate that SCOIGET reduces error metrics by 30–80% while achieving superior pattern congruence.

In addition to its high inference accuracy, the spatial CNV features derived from SCOIGET were also evaluated for downstream clustering applications. Using the Silhouette Score as a measure of clustering quality, SCOIGET-derived features consistently produced the highest scores—ranging from 0.3525 to 0.4599 in HT225C1 sub-samples, 0.4316 in HT260C1, 0.3973 in HT112C1-U1, and 0.4137 in HT112C1-U2. In comparison, clusters derived from InferCNV exhibited scores ranging from −0.0293 to 0.0976, while CopyVAE showed variable performance across samples (Figure 3B). These results demonstrate that SCOIGET not only enhances the accuracy of CNV estimation but also yields more coherent and well-defined clusters.

Collectively, these findings highlight the effectiveness of SCOIGET in integrating spatial information for CNV inference, leading to enhanced concordance with independent genomic data and robust performance in downstream analyses. This integrated approach provides a powerful tool for elucidating tumor heterogeneity and advancing personalized oncology strategies.

### 3.4. Inferring Clonal Evolution in Colorectal Cancer Progression Through Spatial CNV Analysis

**Figure 4:**
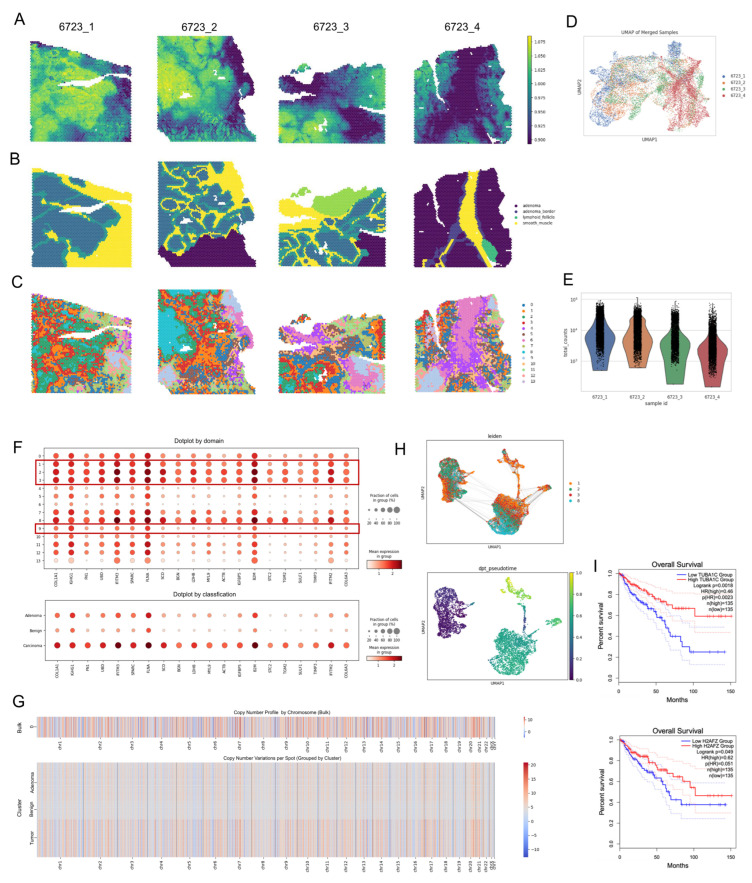
Clonal Evolution in Colorectal Cancer Inferred through Spatial CNV Analysis. (**A**) Spatial CNVs Inferred by SCOIGET. Visualization of copy number variations across the tumor, highlighting regions with distinct CNV patterns. (**B**) Pathologist Annotations. Histological annotations based on H&E-stained images used to validate CNV-based subclones. (**C**) Leiden Clustering of CNV Features. Clustering results showing spatially distinct domains with varying CNV scores. (**D**) UMAP Visualization of Patient Samples. Four patient samples (6723_1, 6723_2, 6723_3 from G2 stage [cancer], and 6723_4 from G1 stage [precancer]) visualized in UMAP space after grouping by tumor stage. (**E**) Total Gene Expression Counts. Bar plot illustrating the total counts of gene expression across the four samples. (**F**) Differential Gene Expression Dotplot. Dotplot showing differentially expressed genes in the 14 domains identified in panel (**C**), grouped into benign, adenoma, and carcinoma categories. (**G**) Chromosome-Level CNV Heatmaps. Heatmaps displaying copy number variations segmented by chromosomes for bulk tumor samples and subgrouped domains (benign, adenoma, carcinoma). (**H**) Identification of Clones and Clonal Trajectories. Four distinct clones (cloneA, cloneB, cloneC, cloneD) identified within carcinoma-associated domains, with UMAP projections and clonal trajectories inferred using PAGA analysis. CloneA and cloneB appear only in the cancerous stage, cloneC spans both G1 and G2 stages, and cloneD marks the transitional zone. (**I**) Survival Analysis of Differential Genes. Survival curves for differential genes TUBA1C and H2AFZ in cloneB, showing significant prognostic differences in CRC patient survival based on TCGA data. The dotted lines represent the 95% confidence intervals for each group.

To investigate tumor evolution, we applied SCOIGET to four 10× Visium CRC samples derived from the same patient’s cecum, including one G1-stage sample (6723_4, TVA subtype) and three G2-stage MSS samples (6723_1, 6723_2, 6723_3). Following integration of basic expression profiles with Harmony, SCOIGET generated spatial CNV scores that delineated tumor boundaries and highlighted malignant potential (Figure 4A). Leiden clustering of these CNV features identified spatially distinct domains corresponding to the tumor’s clonal architecture (Figure 4C). High-CNV-score domains (1, 2, 3, 8) were centrally localized, while intermediate-CNV domains (0, 5, 7, 9, 10, 12, 13) and low-CNV-score domains (4, 6, 11) occupied the periphery. These domains were associated with four subclones: cloneA (domain2), cloneB (domain8), cloneC (domain1), and cloneD (domain3). CloneA and cloneB were exclusive to the cancerous stage, cloneC spanned both precancerous and cancerous phases, and cloneD marked a transitional boundary (Figure 4H).

Pathologist annotations of HE-stained images validated the CNV-based classifications (Figure 4B). UMAP visualization of the four samples by tumor stage confirmed the spatial segregation of cancerous and precancerous regions (Figure 4D), while total gene expression counts across these stages highlighted distinct expression patterns (Figure 4E). Differentially expressed genes among the 14 domains reinforced the CNV-based classifications, with cancerous regions showing elevated expression of CRC-associated markers (Figure 4F). Chromosome-level CNV heatmaps further delineated alterations across tumor subtypes, distinguishing benign, adenoma, and carcinoma regions (Figure 4G).

By integrating chromosome-level CNV profiles and assessing clonal trajectories, SCOIGET pinpointed cloneC as pivotal in transitioning from G1 to G2 stages, suggesting its involvement in early tumor progression (Figure 4H). Furthermore, survival analysis of cloneB-specific genes, such as TUBA1C and H2AFZ, revealed significant prognostic differences in CRC patients based on TCGA data (Figure 4I). TUBA1C, which encodes a tubulin alpha chain essential for microtubule dynamics, is overexpressed in colorectal and other cancers. Its elevated expression is associated with poor prognosis, tumor progression, and modulation of the tumor microenvironment, suggesting its potential as both a prognostic biomarker and therapeutic target [35,36]. Meanwhile, H2AFZ, a histone variant involved in chromatin remodeling and transcriptional regulation, plays a critical role in tumorigenesis. Its overexpression has been strongly associated with enhanced tumor aggressiveness, epithelial–mesenchymal transition (EMT), and poor survival outcomes across various cancers, underscoring its importance as a prognostic indicator [37,38].

These findings reinforce the biological significance of these markers and highlight how SCOIGET’s robust CNV estimates can direct downstream functional analyses, ultimately enhancing our understanding of tumor progression and guiding targeted, precision oncology strategies.

### 3.5. SCOIGET Reveals Tumor Evolution Patterns in Prostate Cancer

**Figure 5:**
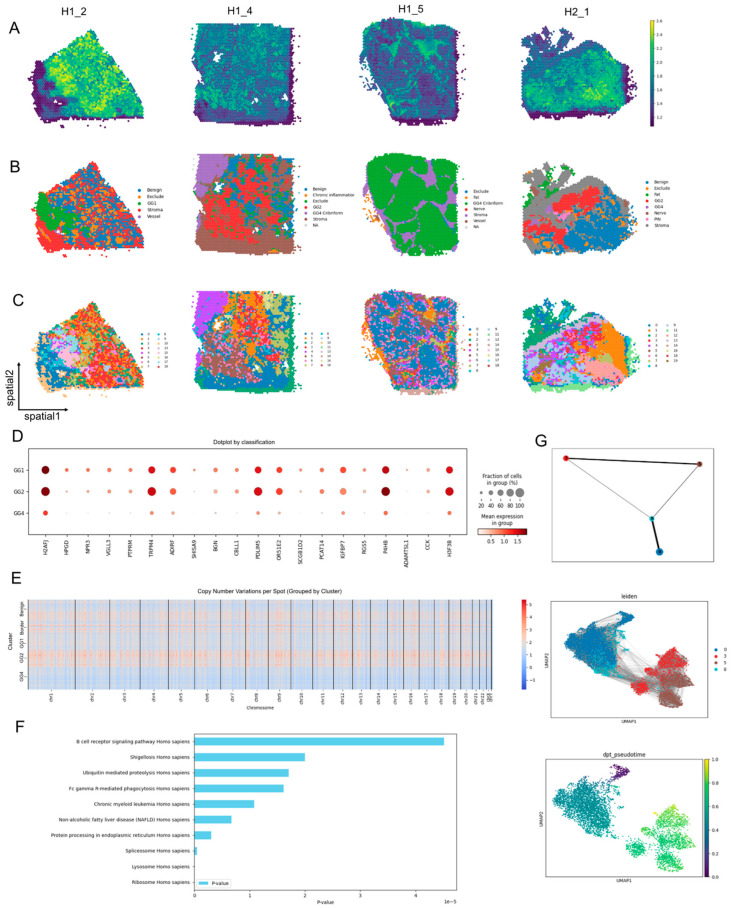
Tumor Evolution Patterns in Prostate Cancer Revealed by SCOIGET. (**A**) Spatial CNVs inferred by SCOIGET. Visualization of copy number variations across tumor regions, capturing distinct CNV patterns in prostate cancer samples. (**B**) Pathologist annotations. HE-stained histological images annotated by pathologists, validating SCOIGET-derived spatial CNV clusters. (**C**) Leiden clustering of CNV features. Spatial domains identified based on CNV features, corresponding to Gleason Grades (GG1, GG2, GG4), border, and benign regions. (**D**) Differential gene expression dotplot. Dotplot showing distinct gene expression profiles across tumor grades, highlighting key genes associated with tumor progression. (**E**) Chromosome-level CNV heatmaps. Heatmaps of copy number variations grouped by chromosome, demonstrating genomic heterogeneity among different Gleason Grades and benign regions. (**F**) Pathway enrichment analysis. Bar plot of enriched pathways among differentially expressed genes across tumor clusters, emphasizing pathways linked to immune regulation and tumor progression. (**G**) Clonal evolution trajectories inferred by PAGA. PAGA-based analysis mapping tumor clonal evolution, identifying cluster 0 as the root node and illustrating the progression and divergence of tumor clones.

To evaluate model’s generalizability, we applied SCOIGET to four Visium PCa samples collected from distinct regions of the same patient. SCOIGET effectively inferred spatial CNV features, identifying both shared and region-specific genomic alterations (Figure 5A). Leiden clustering revealed spatial domains corresponding to Gleason Grades (GG1, GG2, GG4), border regions, and benign areas, aligning with histological classifications and revealing finer structures (Figure 5C). These clustering results were validated by pathologist annotations from HE-stained images, demonstrating strong concordance (Figure 5B).

Further analyses illuminated molecular distinctions between tumor grades. Differentially expressed genes among clusters were enriched in pathways associated with tumor progression and immune modulation, including the B cell receptor signaling pathway, ubiquitin-mediated proteolysis, and chronic myeloid leukemia (Figure 5F). Chromosome-level CNV heatmaps showcased unique and shared alterations across tumor grades, providing detailed insights into genomic heterogeneity (Figure 5E).

Using PAGA, SCOIGET reconstructed clonal evolution trajectories, identifying cluster 0 as the root node and mapping the progression and divergence of tumor clones (Figure 5G). These integrated analyses underscore SCOIGET’s utility in elucidating clonal dynamics and identifying biologically significant features, offering potential insights into therapeutic targets and prognostic biomarkers.

These findings affirm SCOIGET’s ability to resolve tumor evolution within heterogeneous tissue environments. By integrating spatial omics with CNV inference, SCOIGET provides a robust platform for investigating tumor heterogeneity, advancing biomarker discovery, and supporting precision oncology.

### 3.6. Unveiling Colorectal Cancer Heterogeneity at Subcellular Resolution

**Figure 6:**
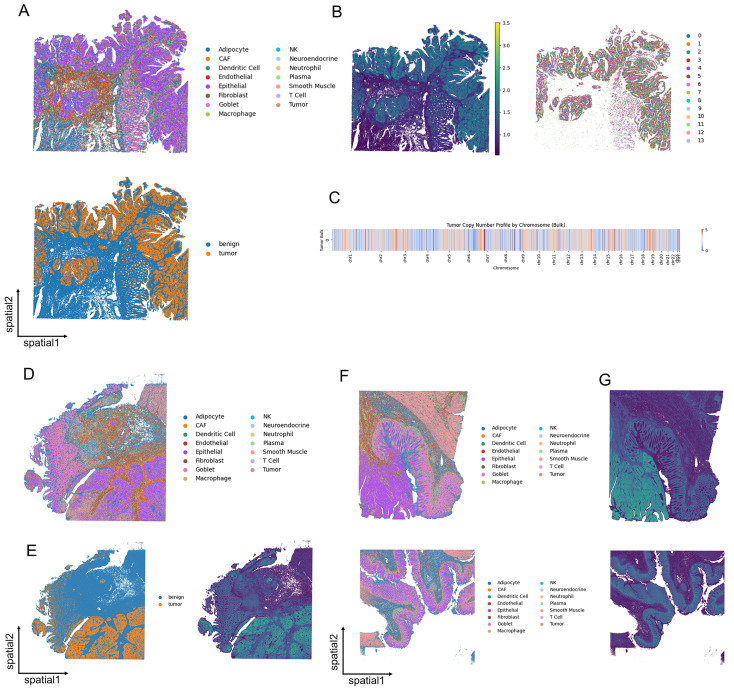
SCOIGET applied to subcellular-resolution CRC spatial omics data. (**A**) Annotation of cell types in the p2 sample, with malignant and benign regions distinguished based on gene expression profiles. (**B**) Spatial CNV inference results for p2, coupled with Leiden clustering of tumor regions. (**C**) Chromosome-level bulk tumor CNV heatmap showcasing inferred genomic alterations. (**D**) Cell type annotations in the p1 sample, highlighting malignant and benign areas. (**E**) Spatial CNV inference results for p1, revealing tumor boundaries and genomic transitions. (**F**) Cell type annotations in the p5 sample, which includes CRC and NAT (normal adjacent tissue) regions. (**G**) Spatial CNV inference results for p5, illustrating tumor and control region differences.

To further validate its versatility, we applied SCOIGET to subcellular-resolution spatial transcriptomics data from three CRC samples (p1, p2, and p5), each collected from a distinct patient. After annotating cell types using established marker genes, we distinguished malignant and benign regions to serve as a reference framework (Figure 6A,D,F). SCOIGET-derived CNV features revealed a strong correspondence between spatially inferred genomic alterations and tumor microenvironment boundaries, effectively highlighting transitions between tumor and non-tumor areas (Figure 6B,E,G).

Chromosome-level CNV predictions at the bulk tumor level further supported these observations, showcasing consistent alterations across spatial regions (Figure 6C). Notably, SCOIGET successfully identified subtle spatial patterns of genomic alterations, even in subcellular-resolution data, offering insights into the spatial and evolutionary heterogeneity of CRC.

These results highlight SCOIGET’s adaptability to high-resolution spatial omics platforms, enabling detailed exploration of tumor microenvironment dynamics and evolution at single-cell resolution. Its ability to capture fine-grained spatial evolutionary patterns underscores its potential for broader applications, offering new avenues for interpreting tumor heterogeneity and progression.

## 4. Discussion

In this study, we introduced SCOIGET, a novel graph-based framework designed to infer copy number variations (CNVs) and map clonal architecture from spatial transcriptomics data. By uniquely integrating spatial information through graph neural networks (GNNs), SCOIGET provides a more nuanced understanding of tumor heterogeneity than traditional single-cell methods. We demonstrated its effectiveness in identifying spatial domains, inferring evolutionary trajectories, and its versatility across different cancer types (CRC and PCa) and technology platforms (10× Visium and Visium HD).

### 4.1. Comparison with Existing Methods

A key innovation of SCOIGET is its ability to leverage spatial coordinates, addressing a major limitation of existing CNV inference tools developed for dissociated single-cell RNA-sequencing (scRNA-seq). While powerful tools like InferCNV [11], CopyKAT [12], SCEVAN [13], and CopyVAE [14] are foundational for clonal analysis, they are spatially unaware. By treating cells as independent entities, they cannot model the critical tissue-level interactions and spatial dynamics that drive tumor evolution, which is the primary strength of our GNN-based approach.

When compared to other spatial methods, SCOIGET occupies a distinct and necessary niche. The state-of-the-art tool, CalicoST [17], provides remarkable allele-specific resolution by integrating B-allele frequency (BAF) from SNPs. This allows it to detect complex genomic events, such as copy-neutral loss of heterozygosity (CNLOH) and mirrored subclonal CNVs, which are “invisible to total copy number analysis” like ours.

However, this power comes at a significant cost in accessibility and computational overhead.

1. Input Requirements: CalicoST fundamentally requires allele-specific SNP counts ($Y^{0}$ and $D^{0}$) derived from raw BAM files. This makes it incompatible with probe-based technologies (e.g., Visium CytAssist for FFPE) which “do not sequence SNPs”, as well as many publicly available datasets that only provide gene expression matrices. SCOIGET’s ability to operate directly on standard gene expression matrices grants it far broader applicability.
2. Computational Speed: CalicoST’s statistical optimization model is computationally intensive, with a reported “runtime between 2 and 8 h”. In contrast, SCOIGET’s deep-learning framework is highly scalable, completing its analysis in approximately 35 min on a comparable dataset.

Thus, SCOIGET fills a critical gap: it provides a fast, scalable, and widely applicable framework for spatial CNV analysis that trades the allele-specific resolution of CalicoST for speed and usability on the most common spatial transcriptomics data types.

### 4.2. Clinical Implications and Future Directions

The ability to spatially resolve clonal architecture has direct clinical implications. For instance, SCOIGET’s spatial CNV maps could guide targeted biopsies to ensure the most aggressive or therapy-resistant clones are sampled, overcoming the spatial biases inherent in bulk sequencing. Furthermore, by identifying spatially defined subclones associated with poor prognosis (as shown in our survival analysis), our method could serve as a valuable tool for patient stratification and predicting therapy response.

Future research should focus on integrating multi-omics data. While SCOIGET provides a robust framework, incorporating spatial genomics, epigenomics, or proteomics would undoubtedly improve the accuracy of its predictions. Expanding the GNN features to explicitly model tumor–immune and tumor–stromal interactions also represents a promising avenue for delineating the complex tumor microenvironment.

### 4.3. Limitations of the Study

Despite its promising results, SCOIGET has several key limitations that must be acknowledged. First, as noted by reviewers, inferring CNVs solely from transcriptomic data is an inherent challenge. Gene expression is influenced by numerous confounding factors beyond copy number. As other researchers have noted, it can be “difficult to determine whether an observed gene expression change is a result of CNVs” or other causes, such as “chromatin accessibility and transcription factor binding”. Therefore, the ‘apparent CNVs’ identified by SCOIGET should be interpreted as functional, CNV-related expression signatures rather than a direct, physical measurement of DNA alterations.

Second, as a total copy number method, SCOIGET is “blind” to the allele-specific events like CNLOH that CalicoST is designed to find.

Third, SCOIGET’s normalization strategy relies on the presence of diploid (normal) cells in the sample to establish a baseline. This is a common requirement for unsupervised tools, including CopyVAE (which “depends heavily on the accurate identification of diploid cells”) and InferCNV (which requires a “reference population of normal cells”). In samples of very high tumor purity where no normal cells are present, the model’s accuracy may be reduced.

Nonetheless, SCOIGET’s demonstrated performance underscores its potential as a transformative and practical tool for cancer research, providing a rapid and scalable solution for exploring the spatial-genomic landscape of tumors.

## 5. Conclusions

In this study, we introduced SCOIGET, an innovative graph-based framework designed for the spatially contextualized inference of copy number variations (CNVs). By uniquely integrating spatial omics data with graph neural networks and attention mechanisms, our model accurately identifies and characterizes spatial copy number features. A key strength of SCOIGET is its ability to construct detailed representations of tumor evolution, effectively capturing intratumor heterogeneity and clonal dynamics at both cellular and subcellular resolutions.

Our comprehensive evaluations demonstrated that SCOIGET consistently outperforms existing methods across multiple datasets, showcasing its robustness and versatile applicability to diverse spatial omics platforms and cancer types. Consequently, SCOIGET provides a powerful tool for a range of critical downstream analyses, including tumor subclone identification, spatial clustering, and the inference of evolutionary trajectories. Ultimately, our work establishes a robust computational foundation for dissecting spatial genomic landscapes, offering new avenues to advance precision oncology strategies and deepen our understanding of cancer evolution.

## Data Availability

The study uses publicly available spatial transcriptomics data. CRC Visium data is retrieved from HTAN WUSTL and Vanderbilt Atlas by HTAN DCC Portal (https://data.humantumoratlas.org/, accessed on 15 Feburary 2024). CRC VisiumHD data is retrieved from 10× genomics (https://www.10xgenomics.com/products/visium-hd-spatial-gene-expression/dataset-human-crc/, accessed on 10 July 2024). PCa Visium data is retrieved from Mendeley Data (https://doi.org/10.17632/svw96g68dv.1, accessed on 20 September 2024). The code is publicly available at https://github.com/YukiZH/SCOIGET, accessed on 20 November 2025, under MIT license.

## Abbreviations

The following abbreviations are used in this manuscript:

CNV	Copy Number Variation
CRC	Colorectal Cancer
EMT	Epithelial–Mesenchymal Transition
GAT	Graph Attention Network
GG	Gleason Grade
GNN	Graph Neural Network
HMM	Hidden Markov Model
HMRF	Hidden Markov Random Field
HLA	Human Leukocyte Antigen
k-NN	k-Nearest Neighbors
MSE	Mean Squared Error
MSS	Microsatellite Stable
NGS	Next-Generation Sequencing
OS	Overall Survival
PAGA	Partition-based Graph Abstraction
PCa	Prostate Cancer
PCA	Principal Component Analysis
PyG	PyTorch Geometric
SCOIGET	Spatial Copy Number Inference by Graph on Evolution of Tumor
scRNA-seq	Single-cell RNA sequencing
ST	Spatial Transcriptomics
TCGA	The Cancer Genome Atlas
TVA	Tubular Villous Adenoma
VAE	Variational Autoencoder
WES	Whole-Exome Sequencing

## Appendix A

### Appendix A.1. Details in Dataset Construction

We employed four spatial transcriptomics datasets, comprising three colorectal cancer (CRC) cohorts and one prostate cancer (PCa) cohort.

Validation Dataset (Figure 3)
  • Source: HTAN-WUSTL atlas [18].
  • Samples: CRC Visium samples HT260C1, HT112C1 (U1, U2), and HT225C1 (U1–U5).
  • WES Data: Whole-exome sequencing data were included to construct the validation for copy number analysis.
  • Sample Description:
    - HT260C1: Tumor sample.
    - HT112C1 (U1, U2): Two sections of the same tumor.
    - HT225C1 (U1–U5): Multiple regions sampled from one tumor.
Case Study 1 (Figure 4)
  • Source: 2023 Cell publication [19].
  • Samples: Four CRC Visium samples from the same patient (PAT71397).
  • Stages:
    - G1 Stage (AD): Sample 6723_4, identified as a tubular villous adenoma (TVA).
    - G2 Stage (IIIB): Samples 6723_1, 6723_2, 6723_3, classified as microsatellite stable (MSS) stage IIIB tumors.
  • Specimen Location: Cecum region.
Case Study 2 (Figure 5)
  • Source: 2022 Nature study [3].
  • Samples: Four prostate cancer Visium samples (H1_2, H1_4, H1_5, H2_1) from a single patient.
Case Study 3 (Figure 6)
  • Source: Publicly available 10× Genomics and Zenodo repository [20].
  • Samples: Three CRC Visium HD samples (p1, p2, p5).
  • Processing:
    - Samples p1 and p5 used 8 μm × 8 μm bins.
    - Sample p2 underwent Stardist cell segmentation on HE-stained images, supplemented by pathologist-provided annotations.

**Table A1:** Descriptions of datasets.

Figure	Platform	Cancer Type	Source Atlas	Patient ID	Location	Type	Grade	Stage	Gender	Age
Figure 3	10× Visium	CRC	HTAN_WUSTL	HT260C1	/	Metastasis	/	/	/	/
	10× Visium	CRC	HTAN_WUSTL	HT112C1	/	Metastasis	/	/	/	/
	10× Visium	CRC	HTAN_WUSTL	HT112C1	/	Metastasis	/	/	/	/
	10× Visium	CRC	HTAN_WUSTL	HT230C1	/	/	/	/	/	/
	10× Visium	CRC	HTAN_WUSTL	HT225C1	/	/	/	/	/	/
Figure 4	10× Visium	CRC	CRC_ST	PAT71397	Cecum	MSS	G2	IIIB	M	61
	10× Visium	CRC	CRC_ST	PAT71397	Cecum	MSS	G2	IIIB	M	61
	10× Visium	CRC	CRC_ST	PAT71397	Cecum	MSS	G2	IIIB	M	61
	10× Visium	CRC	CRC_ST	PAT71397	Cecum	TA/TVA	G1	AD	M	61
Figure 5	10× Visium	PCa	PCa_ST	H1_2	/	/	/	/	/	/
	10× Visium	PCa	PCa_ST	H1_4	/	/	/	/	/	/
	10× Visium	PCa	PCa_ST	H1_5	/	/	/	/	/	/
	10× Visium	PCa	PCa_ST	H2_1	/	/	/	/	/	/
Figure 6	Visium HD	CRC	Visium HD	p2	Sigmoid	/	/	/	M	60
	Visium HD	CRC	Visium HD	p1	Sigmoid	/	/	IIA	F	72
	Visium HD	CRC	Visium HD	p5	Sigmoid	/	/	IVA	F	58

### Appendix A.2. Details of the SCOIGET Algorithm

Input Data

SCOIGET’s input consists of three key components:

• Node Features: Each spot or cell is represented by either binned gene expression features (feat) or pseudo-copy number profiles (norm_x), depending on the training stage.
• Spatial Graph Structure: The spatial relationships between spots were encoded in an adjacency matrix (graph_neigh), where nodes represent spots or cells, and edges represent spatial proximity based on the tissue’s architecture. This graph structure preserves the spatial context of the data.
• Edge Attributes: Edge weights and probabilities were computed to quantify the similarity between neighboring spots. These attributes were calculated based on distances in the gene expression feature space and transformed using softmax normalization.

Model Architecture

SCOIGET’s model comprises three main components: an encoder, a decoder, and a copy number encoder.

• Encoder: The encoder employs three GAT layers to learn latent representations of the input data. Each layer attends to neighboring nodes in the spatial graph, allowing the model to focus on relevant spatial relationships and capture both local and global patterns. The encoder incorporates multiple attention heads (e.g., eight heads per layer), and ReLU activations are used for nonlinearity. The final layer aggregates the features and outputs the mean (z_mean) and variance (z_var) of the latent representations, with variance constrained to ensure numerical stability.
• Decoder: The decoder reconstructs the original input features from the latent representations. It uses fully connected layers, including an intermediate layer with 128 units, and a final output layer matching the input dimension. ReLU activations introduce nonlinearity, ensuring the model captures complex patterns during reconstruction.
• Copy Number Encoder (CNEncoder): The CNEncoder estimates CNVs from the reconstructed features using a Hidden Markov Model (HMM). This model identifies regions with consistent copy number states, iteratively predicting hidden states that correspond to different copy number levels. The model’s output is adjusted by spatial smoothing, where neighboring spots’ states are averaged, using the edge_index parameter to encode spatial relationships. This spatial smoothing reduces noise and improves the localization of CNVs. The final CNV estimates are normalized, rescaled, and adjusted to ensure consistency across samples. Additionally, a regularization loss term helps prevent overfitting.

Training procedure

The model is trained in two phases:

• First Training Phase: The initial training uses the binned gene expression data and spatial graph constructed from the original features. The model is trained without a validation set to learn initial latent representations and reconstruct the input features. The loss function combines reconstruction loss (mean squared error between the original and reconstructed features) and a Kullback–Leibler (KL) divergence term to regularize the latent space. After training, the CNEncoder estimates pseudo copy numbers using the HMM. These estimates serve as initial CNV predictions.
• Second Training Phase: The pseudo copy numbers obtained from the first phase are incorporated into the node features. A new spatial graph is constructed using these updated features. The model is retrained on the new graph, this time employing a validation set to monitor performance and prevent overfitting. Early stopping criteria may be used based on validation loss. The CNEncoder re-estimates the copy numbers, refining the CNV profiles with the updated model.

### Appendix A.3. Copy Number Estimation and Refinement

Copy number variations (CNVs) are estimated in the SCOIGET framework using CNEncoder, which employs an HMM to capture sequential dependencies in genomic data, identifying regions with consistent copy number states.

Hidden Markov Model for CNV Detection

The HMM models genomic bins (group of adjacent genes) with

• States (S): Discrete copy number states, such as deletion ($s_{1}$), normal ($s_{2}$), and amplification ($s_{3}$).
• Observations (O): Reconstructed gene expression levels per bin.
• Initial State Probabilities (π): Probabilities for starting in each state.
• Transition Probabilities (A): Probabilities of transitioning between states in adjacent bins.
• Emission Probabilities (B): Likelihood of observing a particular expression level given a state, modeled as Gaussian distributions $N\left(\mu_{i},\sigma_{i}^{2}\right)$.

Estimation Process

• Initialization: Parameters π, A, $\mu_{i}$, and $\sigma_{i}^{2}$ are initialized based on biological expectations, favoring state persistence.
• Expectation-Maximization (EM) Algorithm: E-step computes posterior probabilities of the states given the observations using the forward-backward algorithm. M-step updates the HMM parameters to maximize the expected log-likelihood of the data.
• Viterbi Decoding: Determines the most probable sequence of hidden states $\left\{q_{t}\right\}$, assigning a copy number state to each bin.

Normalization and Adjustment

The following is carried out to account for technical variability and ensure comparability:

• State Mapping: Map hidden states to copy number values $c_{t}$ (e.g., $c_{t}=1$ for deletion, $c_{t}=2$ for normal, $c_{t}=3$ for amplification).
• Copy Number Adjustment:
  (A1) $$\tilde{c}t=\frac{c_{t}}{\sum tc_{t}}\sum_{t}o_{t}$$
• Spot Normalization:
  (A2) $$\tilde{c_{t}^{\mathrm{norm}}}=\frac{\tilde{c_{t}}-\min\left(\tilde{c}\right)}{\max\left(\tilde{c}\right)-\min\left(\tilde{c}\right)}$$

Normalizes the adjusted copy numbers across all spots.

### Appendix A.4. Integration into the GNN

In the second training phase, the normalized copy number estimates $\tilde{c_{t}^{\mathrm{norm}}}$ are incorporated into the node features of the graph neural network. This enriched feature set allows the model to learn refined spatial-genomic patterns, enhancing CNV detection accuracy and improving the identification of spatial domains within the tissue.

• Regularization

A regularization term is added to the loss function to promote smoothness of copy number estimates across neighboring spots:

(A3) $$L\mathrm{reg}=\lambda \mathrm{reg}\sum_{\left(i,j\right)\in E}w_{ij}{\left(\tilde{c_{i}^{\mathrm{norm}}}-\tilde{c_{j}^{\mathrm{norm}}}\right)}^{2}$$

where E is the set of edges in the spatial graph, $w_{ij}$ are the edge weights based on spatial proximity or expression similarity, $\lambda_{\mathrm{reg}}$ controls the strength of regularization.

### Appendix A.5. Evaluation Metrics

The following metrics are used to comprehensively assess the performance of SCOIGET and the contrast algorithms:

Mean Squared Error (MSE): Measures the average squared difference between predicted and true copy number values (WES baseline):
  (A4) $$\mathrm{MSE}=\frac{1}{N}\sum_{i=1}^{N}{\left(y_{i}-\hat{y_{i}}\right)}^{2},$$
  where $y_{i}$ is the true value, and $\hat{y_{i}}$ is the predicted value.
Cosine Similarity: Measures the similarity between two vectors, treating CNV profiles as high-dimensional vectors:
  (A5) $$\mathrm{Cosine}\;\mathrm{Similarity}=\frac{\sum_{i=1}^{N}y_{i}\hat{y}i}{\sqrt{\sum i=1^{N}y_{i}^{2}}\cdot \sqrt{\sum_{i=1}^{N}\hat{y_{i}^{2}}}}$$

A value closer to 1 indicates higher similarity.

Euclidean Distance: Quantifies the straight-line distance between predicted and true CNV values:
  (A6) $$\mathrm{Euclidean}\;\mathrm{Distance}=\sqrt{\sum_{i=1}^{N}{\left(y_{i}-\hat{y_{i}}\right)}^{2}}.$$
Manhattan Distance: Calculates the absolute distance between predicted and true CNV values:
  (A7) $$\mathrm{Manhattan}\;\mathrm{Distance}=\sum_{i=1}^{N}\left|y_{i}-\hat{y_{i}}\right|.$$
Silhouette Score: Evaluates clustering performance by measuring intra-cluster cohesion and inter-cluster separation:
  (A8) $$S\left(i\right)=\frac{b\left(i\right)-a\left(i\right)}{\max\left(a\left(i\right),b\left(i\right)\right)},$$
  where $a\left(i\right)$ is the average distance from point i to all other points within the same cluster, $b\left(i\right)$ is the average distance from point i to all points in the nearest neighboring cluster. The silhouette score ranges from −1 to 1, with higher values (closer to 1) indicating well-defined and well-separated clusters.

## Appendix B

### Appendix B.1. Implementation Details for Baseline Methods

To ensure fair comparison and reproducibility, we standardized the execution environments and parameter settings for all baseline methods. Table A2 provides the exact configurations used in this study.

**Table A2:** Detailed Parameter Settings for Baseline Methods.

Method	Software/Environment	Key Parameters and Settings
InferCNV [11]	R package v1.14.2	cutoff = 0.1 (optimized for 10× Genomics sparse data), denoise = TRUE, HMM = TRUE, cluster_by_groups = TRUE.
CopyKAT [12]	R package v1.1.0	KS.cut = 0.1 (segmentation sensitivity), ngene.chr = 5, win.size = 25, distance = “euclidean”, genome = “hg20”.
SCEVAN [13]	R package v1.0.3	beta_vega = 0.5 (segmentation granularity), SUBCLONES = TRUE, FIXED_NORMAL_CELLS = FALSE, SCEVANsignatures = TRUE, organism = “human”.
CopyVAE [14]	Python ≥ 3.8	dim_model = 32, layer_encode = 2, layer_decode = 2, epoch = 50, patience = 5.

### Appendix B.2. Model Stability Analysis

To assess the robustness of SCOIGET’s two-stage training process against initialization variability, we conducted a sensitivity analysis. We trained the model on the simulated dataset using five distinct random seeds (42, 123, 2024, 7, 99). As shown in Table A3, the model exhibited exceptional stability across all runs, with negligible variance in both error and similarity metrics. These results confirm that SCOIGET’s performance is robust and reproducible, independent of random initialization.

**Table A3:** Model Stability Statistics across 5 Independent Runs.

Metric	Mean	Standard Deviation (SD)	Min	Max
Mean Squared Error	0.0320	0.0002	0.0316	0.0323
Cosine Similarity	0.6281	0.0008	0.6270	0.6293
